# Resurfacing shoulder hemi arthroplasty in ballistic injuries. A case report

**DOI:** 10.1016/j.ijscr.2019.10.027

**Published:** 2019-10-22

**Authors:** K. Amri, M.A. Chefi, T. Znagui, A. Rafrafi, S. Saadi, L. Nouisri

**Affiliations:** a Military Hospital of Instruction of Tunis, Tunisia; b University of Tunis El Manar, Faculty of Medicine of Tunis, Tunisia

**Keywords:** Shoulder, Ballistics, Arthroplasty, Case report

## Abstract

**Introduction:**

Ballistic traumatology of the shoulder joint is uncommon. Usually, it is associated to comminuted fractures of the upper humerus. Total shoulder arthroplasty has been proposed to restore a good function. The indication of resurfacing hemi arthroplasty still controversial and is not commonly reported after ballistic traumatology.

**Presentation of case:**

We report the case of a 26-year-old soldier victim of a gunshot of the left shoulder entailing a comminuted complex fracture of the upper extremity of the humerus. After shoulder immobilization of 3 months joint mobility was very limited. Radiographs showed bone malunion with a total loss of the hemispherical form of the humeral head. Patient has undergone a resurfacing hemi arthroplasty. Surgery outcome was good. At five years of follow-up, there were no major functional complaints. Clinical and radiological result was satisfactory with a good shoulder mobility.

**Discussion:**

Shoulder arthroplasty provided good functional outcome in case of post fracture sequelae. Difficulty was to choose between total arthroplasty and hemi arthroplasty. In young and active patients, authors disapproved total arthroplasty in post traumatic gleno-humeral arthrosis to prevent revision difficulties especially after glenoid erosion.

**Conclusion:**

Although follow-up still be limited, the indication of shoulder hemi arthroplasty was appropriate regarding functional outcome and patient satisfaction. Further clinical and radiological supervision still necessary to detect glenoid erosion.

## Highlights

• Ballistic tramatology in the upper humerus extremity region presents challenging management difficulties.
• Resurfacing shoulder hemi arthroplasty in ballistic injuries is not commonly reported.
• The indication of resurfacing shoulder hemi arthroplasty was appropriate regarding good functional outcome.

## 1 Introduction

Since the revolution of 2011, the incidence of ballistic injuries became less uncommon in Tunisian Hospitals. Limbs location are rarely life threatening but could be the cause of serious functional disabilities. In the shoulder, difficulties of surgical management results essentially because of the anatomical complexity and the variety of lesions patterns. Usually, Shoulder hemi arthroplasty (SHA) is indicated in patients with intact rotator cuff muscles and glenoid. As ballistic injuries are often associated to an important soft tissue and muscle damages, SHA after ballistic traumatology is not commonly reported.

We present a case of SHA in a patient presenting humeral head malunion after a ballistic injury causing a comminuted fracture of the upper extremity of the humerus. In this case the limited shoulder mobility was related to the pain from the joint incongruity and not muscular damage. We showed that SHA in ballistic injuries could be performed with satisfactory result.

This work has been reported in line with the SCARE criteria [9].

## 2 Case report

**Fig. 1:**
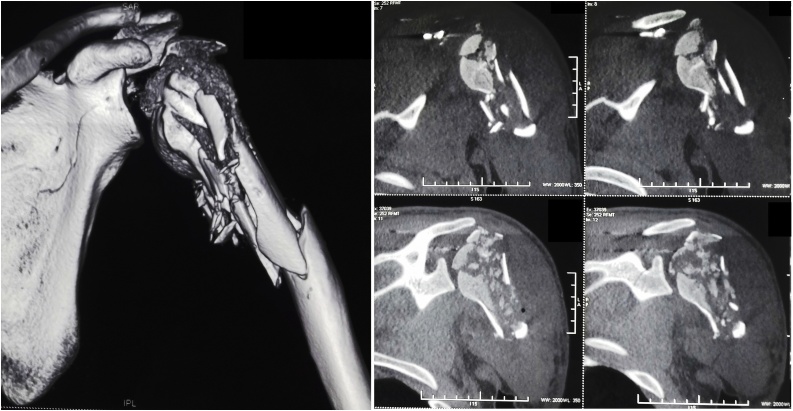
CT scan showing the comminuted fracture of the upper extremity of the humerus.

**Fig. 2:**
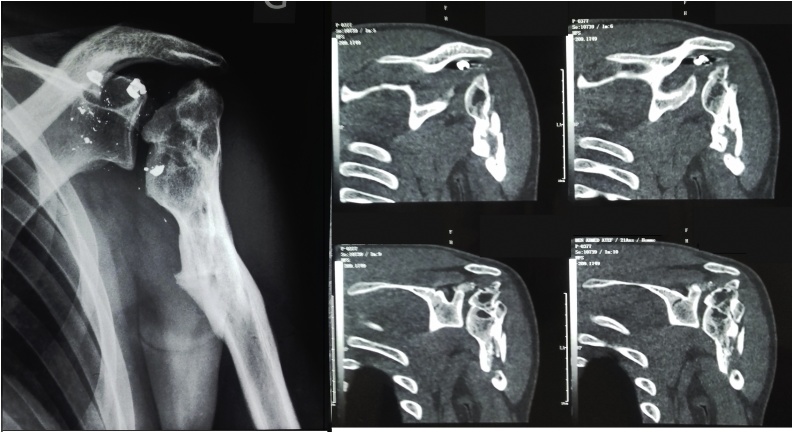
Standard radiography and CT scan showing bone malunion with a total loss of the hemispherical form of the humeral head.

**Fig. 3:**
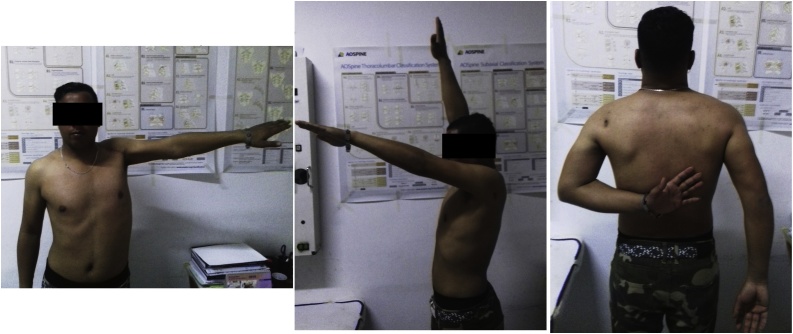
Shoulder active mobility, 2 months after surgery.

**Fig. 4:**
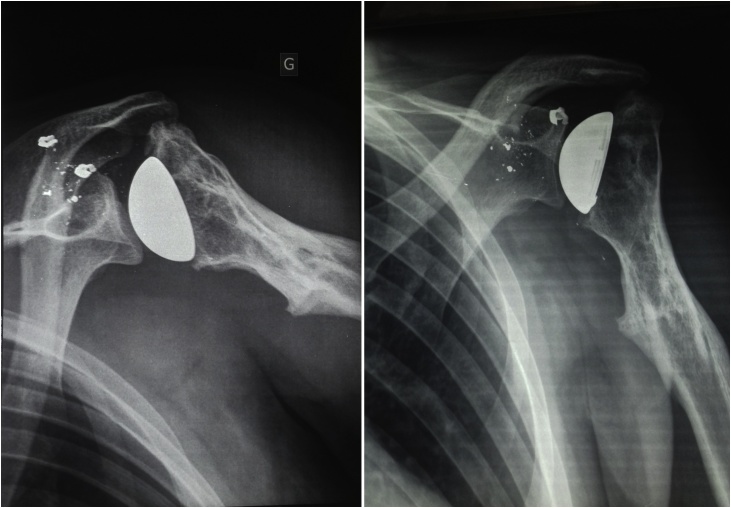
Two months radiographs showing a well positioned implant but a malunion of the greater tubercle.

A 26-year-old soldier, with no past medical history, was shot on the left shoulder on February 2013 in a terrorist attack. The patient was carried from battlefield after 5 h where conditioning and first aid had been done. First examination at emergency room finds a conscious patient with stable hemodynamic and respiratory functions but presenting a total functional disability of the left shoulder. Local examination showed the entry point as a 3 cm jagged-edged wound on the posterior side of the shoulder. Blood and soil were covering the wound. Neuro-vascular examination showed a good radial pulse and no neurological damage. There was no exit point. Computed tomography of the shoulder showed a comminuted complex fracture of the upper extremity of the humerus (Fig. 1). Prophylactic antibiotics were administrated in the emergency room: Penicillin G (2 g intravenously) and Gentamycin (160 mg intramuscular). The patient was conducted to operating room and had undergone an abundant cleaning with debridement of devitalized muscle and soft tissue. Wound bandage was performed without suture. Shoulder immobilization was performed with a U-slab cast on the upper arm in addition of a collar and cuff. Follow up showed total wound healing after one month. Radiographs performed at 4 months showed bone malunion with a total loss of the hemispherical form of the humeral head (Fig. 2). Shoulder mobility was very limited and rehabilitation started in the third month. After 6 months of intensive physiotherapy, clinical outcome was poor and joint mobility was very limited: 5° of external rotation, 10° of abduction and 10° of forward elevation. Shoulder examination was very difficult because mobilization was very painful. Thus, we opted for shoulder examination under Intescalene brachial plexus block. We noted an important increase of passive mobility: 25° of external rotation, 80° of abduction and 90° of forward elevation. Surgery was indicated and discussed with the patient; hence a resurfacing SHA with an Arrow resurfacing cup using a deltopectoral approach was performed in January 2014. Surgery outcome was good and shoulder rehabilitation began the second post operative day. Two months after the surgery, the active mobility was: 30° of external rotation, 90° of abduction, 100° of forward elevation, and internal rotation to the level of L2 (Fig. 3). At five years of follow-up, there were no functional complaints except some moderate pain when abduction exceeds 80°. Patient was satisfied with the shoulder mobility which was the same as 2 months post operative. Radiographs showed a well positioned implant but a hypertrophic union of the greater tubercle which come in conflict with the acromion especially in abduction (Fig. 4). Revision surgery consisting of a tuberoplasty was indicated but refused by the patient. He affirmed that pain is moderate and he was satisfied with the functional outcome.

## 3 Discussion

In ballistic traumatology, injuries may range from superficial laceration to serious lesions including complex fractures, neurovascular lesions, amputation and death. The shoulder location presents several problems essentially the closeness of neurovascular structures and the high incidence of post traumatic joint stiffness. Joint involvement, especially the upper humerus extremity fractures, present very challenging problems. In fact, treatment may vary from non operative to shoulder arthroplasty in case of complex fractures. Good initial management is necessary for better outcome. Hence, early sterile dressing over the wound and early initiation of antibiotics could prevent infections [1]. In operating room, excision should start from the skin wound to the muscle. Viability could be appreciated on texture, bleeding and the ability of contraction. Abundant wash with physiological saline solution should be efficient. Initially, wounds should be left open and covered by sterile dressing and secondary closure could be performed from the fifth day [1]. Although fracture immobilization is necessary, it presents some technical problems for the upper extremity of the humerus. External fixation and splintage are usually used. External fixation was recommended by many authors, but some studies found that splintage in the upper humerus extremity could offer better result and fewer complications [2]. In our case, immobilization was performed with a U-slab cast on the upper arm in addition of a collar and cuff. Often arthroplasty is indicated in cases soft tissue, mainly muscles, were assessed undamaged. For our case, we believed that the problem was the pain from the joint incongruity and not a muscular damage. Thus, arthroplasty was indicated. In fact, it was reported that shoulder arthroplasty provided good functional outcome in case of post traumatic gleno-huméral arthrosis [3]. The difficulty of our case was to choose total arthroplasty or hemi arthroplasty. In fact, in young and active patients, some authors disapprove shoulder total arthroplasty in post traumatic arthrosis to prevent revision difficulties [4]. Besides, in case of deficient glenoid bone capital, it seems difficult to execute glenoid replacement [4]. For our patient, young age, high activity and the less invasive surgical technique lead us to recommend SHA. Besides, the vicious humeral union and deformation motivate us to recommend a resurfacing SHA. We looked in literature for studies analyzing longevity of the prosthesis and the functional outcome. A study analyzing prosthesis longevity in a group of active young patients showed superiority of total shoulder arthroplasty over SHA: 84% versus 73% at 15 years of follow up. But the studied group of patients was heterogenic and arthroplasty performed for post traumatic arthrosis represented only 32% of etiologies [5]. Concerning functional outcome, some authors reported a slight superiority with total arthroplasty [6]. Another studies revealed similar result between the two techniques [7]. For our patient, at 5 years of follow up, there was an important pain relief. Although shoulder mobility did not return to normal amplitudes, it was remarkably greater and patient was satisfied with the result. Radiographic evaluation showed a resurfacing humeral head in place and no glenoid wearing. However, follow up still be limited in our case.

Finally, we looked in literature for similar cases. To our knowledge, this is the first use of resurfacing SHA after ballistic injury. However, Erceg et al. [8] reported a case of SHA in a 26-year-old soldier after war injury. Unlike our case, the result was poor with a limited shoulder mobility: 15° of active abduction, 50° of passive abduction and 60° of forward elevation. This could be explained as a result of the important muscle damages.

## 4 Conclusion

This case showed management difficulties of ballistic tramatology in the upper humerus extremity region. Fracture management still controversial. It depends essentially on fracture type, soft tissue integrity and the surgeon habits. In this case, the indication of shoulder hemi arthroplasty was made carefully and after patient agreement. Although follow-up still be limited, we believe that indication was appropriate regarding good functional outcome and patient satisfaction. Further clinical and radiological supervision still necessary to detect glenoid erosion.

## Sources of funding

We declare there is no sources of funding for this research.

## Ethical approval

This case report is exempt from ethical approval in our institution.

## Consent

Written informed consent was obtained from the patient for publication of this case report and accompanying images.

## Author’s contribution

Conceptualisation: Talel Znagui.

Investigation: Abderrazek Rafrafi.

Data acquisition: Sabeur Saadi.

Manuscript preparation: Mohamed Ali Chefi.

Manuscript editing: Khalil Amri.

Manuscript supervision and validation: Lotfi Nouisri.

## Registration of research studies

This case report is not a research study.

## Guarantor

The guarantor of the study: Mohamed Ali Chefi.

## Provenance and peer review

Not commissioned, externally peer-reviewed.

## Declaration of Competing Interest

The authors declare no conflict of interest.
